# Four Requisites in Development

**Published:** 1896-02

**Authors:** M. F. Ault

**Affiliations:** Kokomo, Ind.


THE DENTAL REGISTER.

VOL. L.] FEBRUARY, 1896. [No. 2.

### COMMUNICATIONS.

### Four Requisites in Development.

BY. M. F. AULT, M.D., D.D.S., KOKOMO, IND.

Read before the Tri-State Meeting in Detroit.

*Continued from the January Number, Page* 19.

INFLUENCE UPON FACULTY.

Let us ask next the influence of such regulation upon a faculty. A faculty certainly labors under embarrassment by having no recognized standard. Each State is fixing its own requirements. Each faculty is teaching largely at random. Each member of a faculty is left to his own option in what he teaches. I mean to say that all dental institutions ought to move around a common center. A convention of competent men ought to define the limits of anatomy, physiology, chemistry and materia medica for a dental college; this convention to take our works on operative and mechanical dentistry and select from them the best, giving full credit to the authors of the parts chosen, and this compilation to constitute the *Standard Text-Book* in these branches. Treat other branches the same, then faculties will know what to teach, the student will know what to study, and the national examiners will know what has been taught and studied, and from which source to select proper test-questions. This single standard will give uniformity, equality in recognition and respect, and all move around a national common center, instead of as many centers and standards as we have States and colleges. Under our present plan a member of a faculty would say, "I select what I want to teach, and my students ought to have their final examination based upon the instruction given." Under our present plan the work of one chair may lap over on to the work of another chair, or repeat and antagonize the thought of a preceding lecture.

Under our present plan one department may say, "I make no effort to do certain work. I am to teach thoroughly what I do teach, and let the rest go."

Under our present plan an instructor is as tardy as he wants to be and irregular in keeping appointments, offering as an excuse that the students are glad to get out of it, or that he had other business, thus making college duty secondary to whim and private affairs.

I claim that a member of a faculty, if given a standard course to follow, would talk less, think to the point, be more prompt, and give the learner the closest personal attention, because to show that tact and qualification which is necessary to polish the student and make him feel the force of all that is said in a standard text-book would determine the reputation and value of a teacher before our highest authority. Reputation would not be left to the caprice of a class of students who seek the least resistance and the desire of a teacher to be solid with the class by being called easy. He would say, "Gentlemen, we must all work; faculty and students must all work, because the national center prescribes the amount of work to be taught, and this is to determine my reputation and your graduation. The day of talk, talk about what you please, and as long as you please upon things relevant and irrelevant, irregular as you choose, and keeping students in subjection by boasting and swelling, has ceased. The day of *merit* is at hand for all."

Now, as one engaged and interested in the daily duties of private practice, I will briefly give the dimensions of the mold in which the next graduate should be cast—of course not attempting to state the table of contents for any standard text-book:

(*a*) Arrange so as to eliminate all superfluity. There is too much lecturing.

(*b*) Selection of men who are willing to work more and talk less, and do it without lamenting that their private practice is suffering. I mean the selection of faculty.

(*c*) Whenever the student is well acquainted with the anatomy of the head and internal viscera, by taking the scalpel in his own hand, using his book as a guide, and a demonstrator to make clear that which is not understood;

(*d*) As soon as he has applied himself to the histology of the various tissues by placing his own eye to the microscope, using his book as a guide and a demonstrator to clear up all ambiguity;

(*e*) Whenever he is in full sympathy with the teacher whose knowledge of physiology enables him to reveal the forces of life;

(*f*) As soon as he has seen, touched and tested the qualities of medicines used in dentistry;

(*g*) Whenever he has mastered the chemistry that may have a bearing in dentistry by taking the test-tube in his own hand, with a careful leader to tell what to do and what to expect from the experiment;

(*h*) As soon as he has been placed in full relation with disease about the teeth by the oversight of a careful diagnostician;

(*i*) Whenever he can take instruments and material and prove that dental restoration and dental substitution is a part of his being;

(*j*) And whenever (whether it be one or five years) he proves it all to the satisfaction of a central national authority, he should be graduated, and his diploma will have a value which can never be given by a fickle, sympathetic faculty—can never be given by a State Board, which is very apt to be partial to its own State, but will have the added value of national endorsement.

This being the order, we would no more see the dissecting-room a place of hilarity, irreverence and random cutting. Surrounding a body which was consigned to the tomb probably forty-eight hours before, amid the tokens, tears and agony of that well-known occasion, the student would deport himself as a representative of science, instead of being a barbarian and lost to all decency and sympathy.

This being the order, we would no longer visit laboratories and infirmaries seeing only those present who choose to be there —those that are there fumbling after something they know not what—absence of system, general irritability, the demonstrator late or absent, and the one selected for his place chosen because of excessive bravado, giving his opinions as facts, when, in reality, the big-head is all he has to exhibit.

This being the order, we will never more enter a lecture-room to hear the professor prove that *he* has a good lesson, the student dazed by fluency on ridges and tuberosities, the time wasted, and the learner injured in his effort to carry an indiscriminate mass in memory; but the instructor will guide, and the pupil will prove that *he* is the one that has his lesson.

INFLUENCE UPON THE PROFESSION.

Such a policy would certainly meet the approbation of members of the profession. A plan like this, submitted to a man in private practice, would be judged according to his first training, the bent of mind at the time the question is submitted, his insight into the need of advancement, and his capacity for grasping results. One would say: "That is so far ahead of anything that I had ever considered as practical that it stuns me." Five minutes after he would say: "It is taking root; it grows on me." One day after that: "That is the college; let us incorporate."

No method of college management should be endorsed, unless it can stand the test of common, every-day reasoning, and it requires no strain of the imagination to see that every dentist in practice would decide that that plan will guarantee—

(*a*) Competency in graduates.

(*b*) Extinction of professional harlots.

(*c*) It will limit competition.

(*d*) It will force the college to serve the profession.

(*e*) It will drive the drones from faculties.

(*f*) It will enable us, without blushing, to say Good Repute; and rescue us from the fatal stench which envelops us.

As a man in private practice, I ask whether a private corporation (and many colleges are such only) can conserve their own interests and the profession's interests at the same time? A private corporation wants, on account of its need and its desire for gain, all the students it can induce to come. The profession, on account of its need and desire for efficiency, seeks restriction and the highest development. A private corporation is apt to have a passion for power, and power more often hinges on money than actual service, and we would say nothing about money if it was impossible to compromise.

The profession (in the sense in which I use the term) has a passion for uniformity of service and equality in reputation and respect, also freedom from humiliating and destructive strife.

A private corporation is legally placed at the head, and the replenishing of our ranks is left to its will; it holds the key which locks and unlocks the door through which every entrance is made into our domain. State Boards cannot control, because they are, too often, a part of the corporation.

The profession has the unquestioned right to think, and this inherent right should be freely used in investigating methods, policies and motives before becoming an instrument in securing legal enactment for a private institution, or recommending a student to a college with questionable regulations.

At present, if one hundred young men decide to follow our vocation they expend thirty-two thousand dollars for information before they can feel within the pale of security. Are they safe? Does their information fortify? Is the highest and best vindicated? Or does a retrospection mortify? Now, have I conveyed the idea that our colleges are not performing their function, *i. e.*, furnishing the various communities with men who know they are skilled and without tremor of hand, huskiness of voice or beaded brow can secure confidence—men who can convince an old practitioner by showing diploma that help can be employed without feeling that a burden has been added to the office? When a man employs help he wants to feel a transfer of responsibility, and the presentation of a diploma ought to be a sure guarantee that the required help can be found in the owner of the diploma. I mean, then, to assert (being also able to prove) that our present college system is not performing its function, basing my assertion on, and finding my proof in the self-evident truth that *only thoroughly reliable men, should be holders of diplomas.*

I hold that much more can be added to our professional interests than the usual disbursement of thirty thousand dollars secures. I must earnestly recommend a merit system applied to both student and faculty, and a National Board to sit in judgment. This is perfectly feasible, and all that is required is a voluntary acquiescence on the part of colleges, or for the legislatures to take the entrance and final examinations out of the hands of interested persons. As I write this, a recent New York enactment is handed me. May 11, 1895, the governor approved a law which makes dentistry an integral part of medicine. It provides that after 1897 a full high school certificate, or its equivalent, shall be necessary for admission, and places the entrance examination under the Board of Regents of New York University and final examination under the State Examining Board. I mention this to show that my fourth requisite is not extreme in its requirements as to admission, and leads in the method of determining fitness for graduation.

THE NATIONAL REGENTS OR EXAMINERS.

What principle should govern in creating this body? I think the idea that the profession is supreme to all other interests. Let it be understood that the profession is one thing, and colleges and personal matters are positively other things. Personal interest must be made to serve, and colleges be subordinated to professional management.

The *State associations* belong to the *profession,* and should always be over the *college.* The State association and college should work together—the association governing and the college filling only honorary positions; *i. e.*, the association should furnish the college an opportunity to prove to the profession that there is reason for its existence. This would tend to make associations popular and assert their natural rights, and out of it would spring the influence and power to create and sustain the national authority by popular methods and the democratic principle of governing. To recapitulate—the novice, college, profession, professional, and people constitute the elements of dental experience, and the foregoing comment places the college in the very responsible position of determining the future status of dentistry. A port, if properly fortified, prohibits the landing of an enemy; but if the walls are low and the garrison small and poorly equipped, plunder and devastation are sure to follow. This is the relation of the college to the domain occupied by our brethren. Every one who entered the French pavilion at the Exposition was certainly impressed with "The Guardian Spirit of the Secrets of the Tomb," and each, as he looked upon the powerful arm which embraced the urn of ashes, had his own revery and made his own application. After placing myself in full sympathy with the sentiments of the sculptor, I thought how truly, also, is this a symbol of educational institutions. The guardian spirit is the faculty, the urn the receptacle of God's mysteries; but in this the spirit must lift the lid and invite the inquirer to view the beauties within.

